# A case of tubulointerstitial nephritis and uveitis syndrome accompanied by subclinical choroiditis

**DOI:** 10.1186/s12886-023-03172-0

**Published:** 2023-10-20

**Authors:** Takuya Arita, Kenichi Namba, Daiju Iwata, Kayo Suzuki, Yo Ogino, Kazuomi Mizuuchi, Miki Hiraoka, Nobuyoshi Kitaichi, Susumu Ishida

**Affiliations:** 1 Department of Ophthalmology, Faculty of Medicine and Graduate School of Medicine, Hokkaido University, N-15, W-7, Kita-Ku, Sapporo, 060-8638 Japan; 2 Department of Ophthalmology, Healh Sciences University of Hokkaido, Sapporo, Japan

**Keywords:** Tubulointerstitial nephritis and uveitis, Multimodal imaging, Choroiditis

## Abstract

**Background:**

Tubulointerstitial nephritis and uveitis (TINU) syndrome is an uveits characterized by complications of idiopathic acute tubulointerstitial nephritis, and most cases present only anterior uveitis. We report a case of TINU syndrome in which the presence of choroiditis was revealed by multimodal imaging.

**Case presentation:**

A 12-year-old male visited our hospital with a 6-day history of ocular pain and hyperemia. Conjunctival and ciliary injections, 1 + flare and 3 + cells of anterior chamber inflammation with mutton fat keratic precipitates were observed in both eyes (OU), together with redness and swelling of the optic disc OU. Laboratory tests showed slightly high levels of soluble IL-2R and serum β2 microglobulin and markedly high levels of urinary β2 microglobulin. The diagnosis of probable TINU syndrome was established on the basis of bilateral uveitis and urinalysis results in accordance with a clinical criteria of tubulointerstitial nephritis. With treatment with oral prednisolone (PSL) at 20 mg/day, ocular findings improved, and the dose of PSL was gradually reduced and withdrawn 6 months later. However, 1 month later from the withdrawal, ocular inflammation recurred with the presence of retinal exudates and snowball vitreous opacities in the peripheral retina OU. Fluorescein angiography showed leakages from peripheral retinal vessels and staining corresponding to retinal exudates. Indocyanine green angiography showed hypofluorescent dots scattered over the ocular fundus. Optical coherence tomography revealed the presence of choroidal thickening. Laser speckle flowgraphy color map showed a relatively cooler color. Findings from these multimodal images indicated the presence of subclinical choroiditis; therefore, oral PSL was administered again, and ocular inflammatory findings were improved.

**Conclusions:**

TINU syndrome can exhibit subclinical choroiditis detected with multimodal imaging. Further studies are necessary to determine the frequency of subclinical choroiditis in TINU syndrome.

**Supplementary Information:**

The online version contains supplementary material available at 10.1186/s12886-023-03172-0.

## Background

Tubulointerstitial nephritis and uveitis** (**TINU) syndrome is an uveitis characterized by complications of idiopathic acute tubulointerstitial nephritis. Most cases present only anterior uveitis [1], and some present posterior segment findings, including redness and/or swelling of the optic disc, snowball vitreous opacity, and retinal exudates [2]. However, a few cases of TINU accompanied by choroiditis have been reported [3, 4]. We report a case of TINU syndrome in which the presence of choroiditis was revealed by multimodal imaging, including indocyanine green angiography (ICGA), optical coherence tomography (OCT), and laser speckle flowgraphy (LSFG).

## Case presentation

A 12-year-old male visited our hospital with a 6-day history of ocular pain and hyperemia in the left eye (OS). At the first examination, his best-corrected visual acuity (BCVA) was 1.0 OS. Slit-lamp examination revealed the presence of 2 + flare and occasional cells in the anterior chamber, but funduscopic examination did not detect any inflammatory findings in the posterior segment OS. No abnormalities were found in the right eye (OD). Ten days after the initial visit, ocular inflammatory findings appeared OD. Conjunctival and ciliary injections, 1 + flare and 3 + cells of anterior chamber inflammation, and mutton fat keratic precipitates were observed in both eyes (OU). In addition, redness and swelling of the optic disc were observed OU. The patient underwent routine screening tests including blood tests (complete blood count, biochemical tests, serological reaction tests, etc.), urinalysis, and the chest X-ray to identify the cause of uveitis. Some of the results are shown below. Soluble IL-2R (895; normal range, < 459 U/mL) and serum β2 microglobulin (2.24; normal range, 0.8–1.8 mg/dL) showed slightly high levels, along with markedly high levels of urinary β2 microglobulin (1.85; normal range, 0.03–0.28 mg/L). T-SPOT.TB, treponema pallidum latex agglutination, and rapid plasma reagin test were all negative. Angiotensin converting enzyme was within normal limits. Although renal biopsy was not performed, pediatric nephrologist determined that acute interstitial nephritis was present, and then, together with the ocular findings the diagnosis of probable TINU syndrome was established in accordance with a clinical criteria of tubulointerstitial nephritis. After oral prednisolone (PSL) at 20 mg/day was given, the ocular findings improved, and urinary β2-microglobulin levels also normalized. The dose of PSL was reduced by 2.5 mg every 2 weeks up to 5 mg/day, then by 1 mg every 4 weeks and withdrawn 6 months later.

However, recurrence was observed 1 month after cessation of oral PSL. The redness and swelling of the optic disc OS developed again, and subfoveal serous retinal detachment (SRD) and choroidal thickening OS were observed with OCT (Supplementary Figure). Oral PSL at 20 mg/day was given again, and the ocular inflammation subsided. The dose of PSL was reduced more slowly, i.e., reduced by 2.5 mg every 1 month up to 5 mg/day, then by 1 mg every 1 month, and finally, oral PSL was stopped 1 year and 3 months later.

**Fig. 1:**
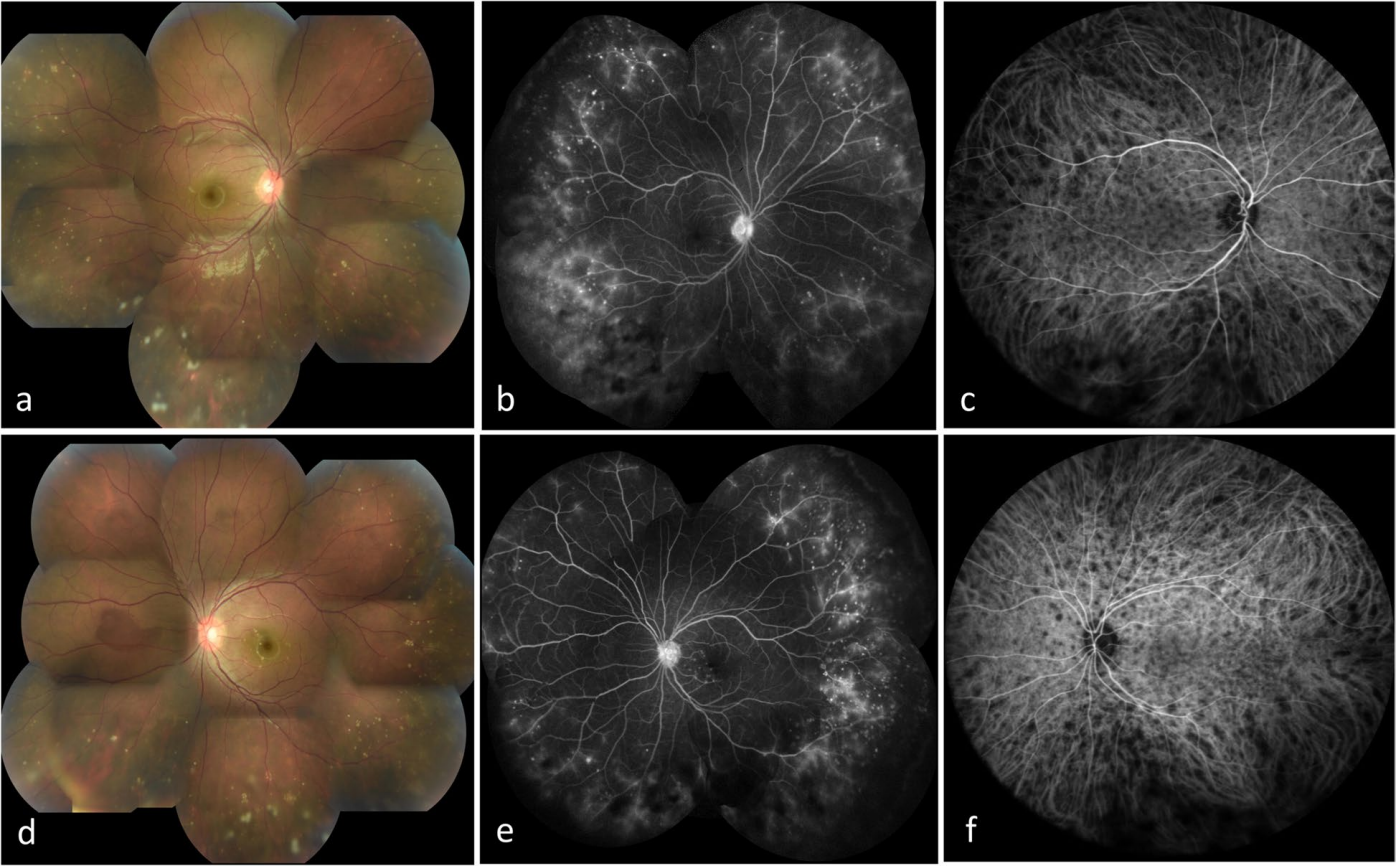
Multimodal imaging (photo, FA, ICGA) at the second relapse. Fundus photographs showing retinal exudates and snowball vitreous opacity around the peripheral retina: **a** (OD), **d** (OS). FA at 10 min showing leakages from peripheral retinal vessels and staining corresponding to retinal exudates: **b** (OD), **e** (OS). ICGA at 1 min showing hypofluorescent dots scattered over the ocular fundus: **c** (OD), **f** (OS)

**Fig. 2:**
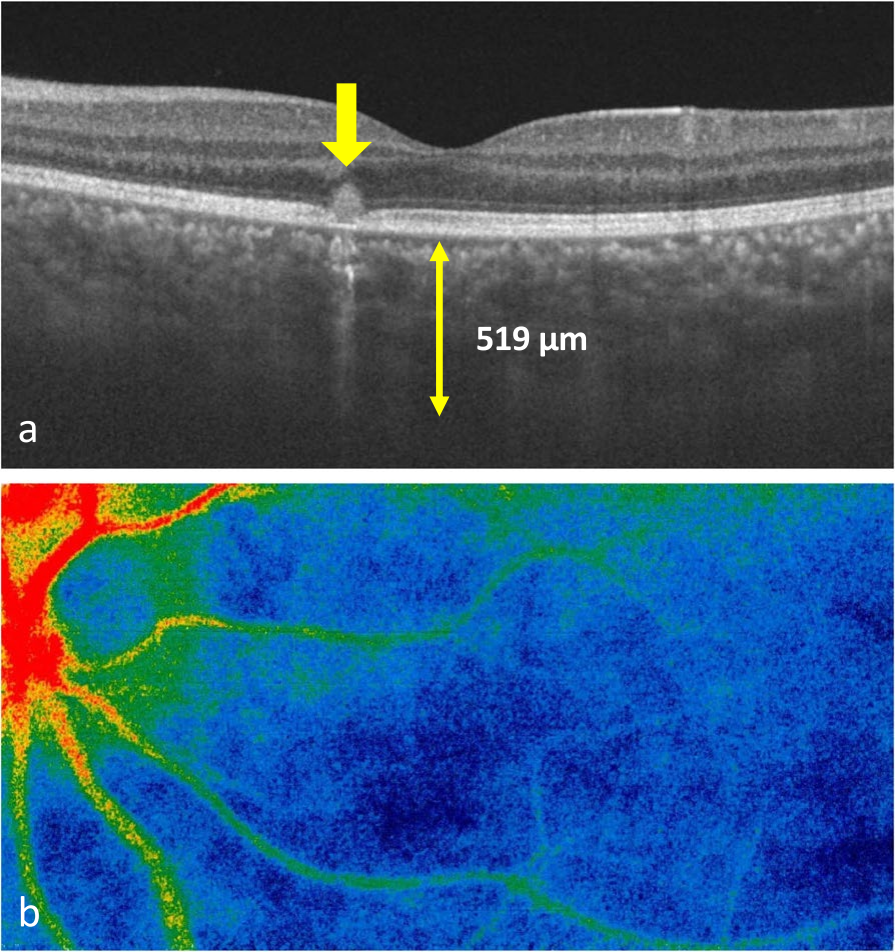
Multimodal imaging (OCT, LSFG) at the second relapse (OS). **a** Swept-source OCT showing the presence of marked choroidal thickenings and a hyper-reflective lesion in contact with the retinal pigment epithelium. **b** LSFG color map showing relatively cooler color, indicating the reduction of choroidal blood flow

The ocular inflammation recurred 1 year and 2 months after the cessation of oral PSL OU. The patient’s BCVA was 1.2 OU. Retinal exudates and snowball vitreous opacities were observed in peripheral retina OU, and retinal exudates were seen even in the posterior pole OS (Fig. 1a, d). Fluorescein angiography (FA) showed leakages from peripheral retinal vessels and staining corresponding to retinal exudates OU (Fig. 1b, e). ICGA showed hypofluorescent dots scattered over the ocular fundus OU (Fig. 1c, f). OCT revealed the presence of choroidal thickenings OU (542 µm OD, 519 µm OS) and a small hyper-reflective lesion in contact with the retinal pigment epithelium OS (Fig. 2a). On LSFG, the color map showed a relatively cooler color, indicating a reduction of choroidal blood flow (Fig. 2b).

**Fig. 3:**
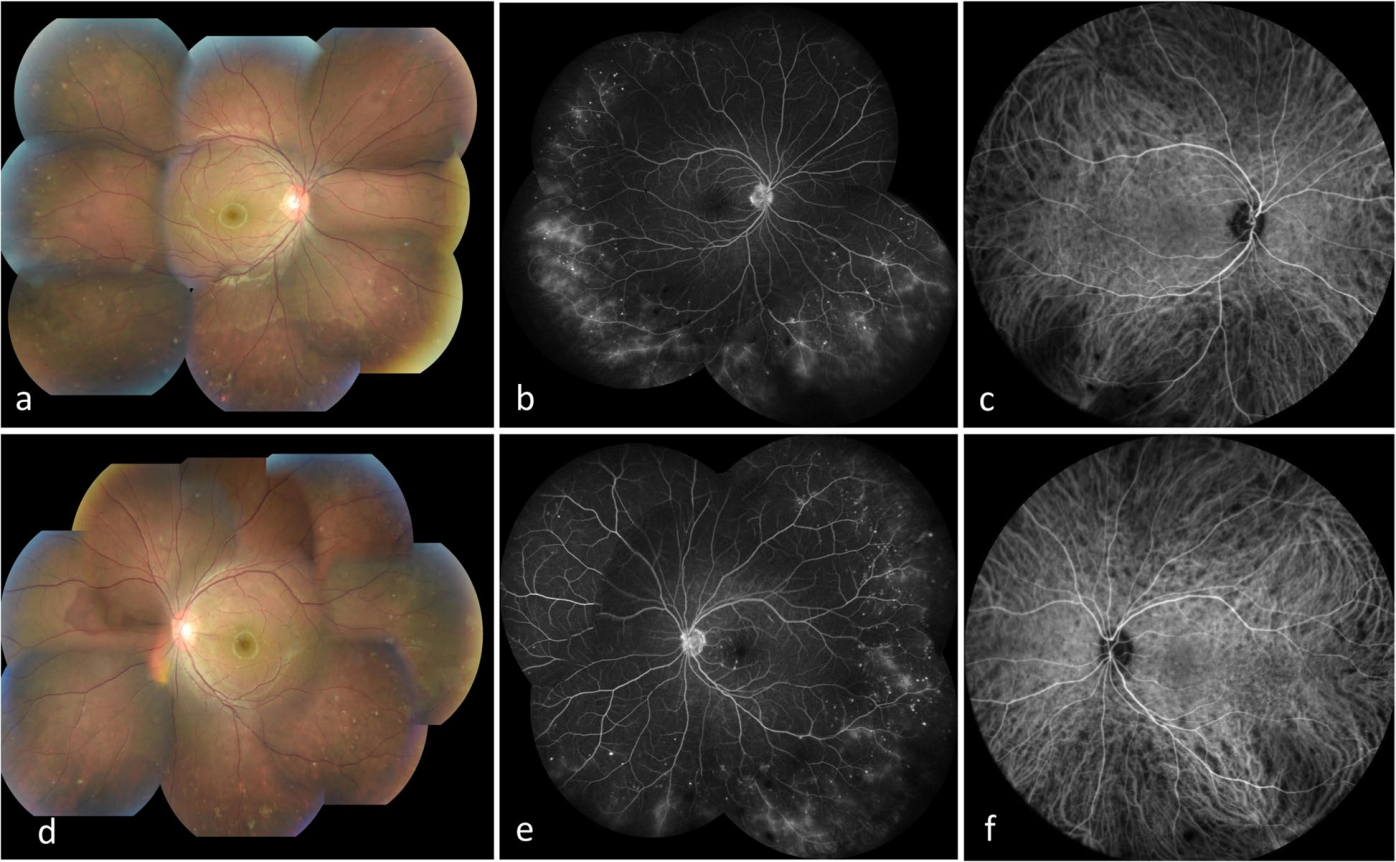
Multimodal imaging (photo, FA, ICGA) after 1 month of PSL at 20 mg/day. Fundus photographs showing a reduced number of retinal exudates and snowball opacities: **a** (OD), **d** (OS). FA at 10 min, showing reduced fluorescence leakage from retinal vessels: **b** (OD), **e** (OS). ICGA at 1 min, showing a reduced number of hypofluorescent dots: **c** (OD), **f** (OS)

**Fig. 4:**
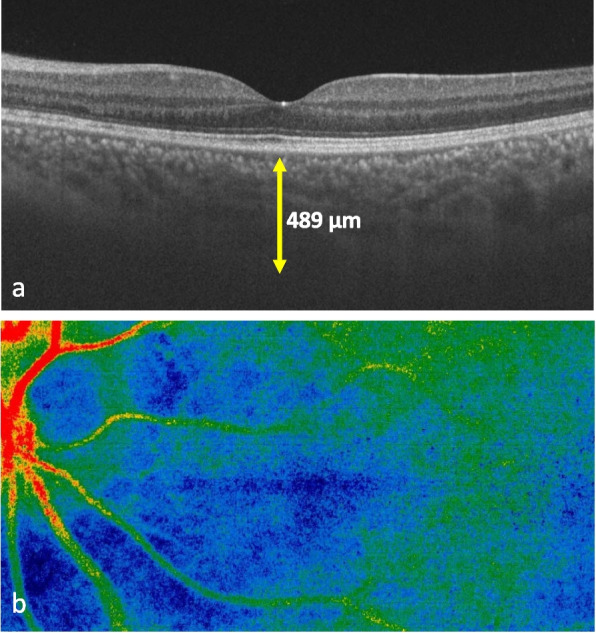
Multimodal imaging (OCT, LSFG) after 1 month of PSL at 20 mg/day. **a** Swept-source OCT showing decreased choroidal thickness and disappearance of hyper-reflective lesion. **b** LSFG color map showing the color change to warmer tones, indicating the recovery of choroidal blood flow

With the findings obtained from multimodal imaging, the presence of a wide range of choroiditis was suspected, whereas the pediatric nephrologist determined that the interstitial nephritis had not recurred because urinary β2 microglobulin and serum creatinine were within normal limits. Therefore, 20 mg of oral PSL was administered again, and 1 month later, the patient’s anterior chamber inflammation almost disappeared OU, and retinal exudates and snowball vitreous opacities diminished (Fig. 3a, d). On FA, fluorescent vascular leakage and staining corresponding to retinal exudates were reduced (Fig. 3b, e). On ICGA, the number of hypofluorescent dots was reduced OU (Fig. 3c, f). On OCT, the choroidal thickness decreased OU (493 µm OD, 489 µm OS), and the hyper-reflective lesion in contact with the retinal pigment epithelium disappeared OS (Fig. 4a). On LSFG, the color map showed that the colors had changed to warmer tones, indicating recovery of choroidal blood flow (Fig. 4b). At present, 10 months later, the dose of PSL has been gradually tapered to 3 mg/day. The patient’s BCVA is 1.2 OD and 1.5 OS, and no anterior segment inflammation is observed. The hypofluorescent dots have disappeared on ICGA, but retinal exudates and snowball vitreous opacities are still observed OU. In addition, choroidal thickening on OCT and fluorescent vascular leakages on FA have also persisted OU. No recurrence of acute interstitial nephritis was seen during the course with normal urinary β2 microglobulin and serum creatinine.

## Discussion and conclusions

Uveitis seen in TINU syndrome is typically characterized by a bilateral, mild anterior presentation, and only 20% of cases present with posterior or pan uveitis [1]. Previously, we reported the ratio of posterior segment findings associated with TINU syndrome as follows: redness and/or swelling of the optic disc, 33%; vitreous opacity, 22%; and retinal exudates, 11% [2].

In this case, in addition to posterior ocular inflammation, such as redness and swelling of the optic disc and retinal exudates, the patient showed subclinical choroiditis that was detected with multimodal imaging (primarily with ICGA, and supplementally with OCT and LSFG), but the symptoms could not be detected by slit-lamp or fundoscopic examination. This case presented SRD at the first relapse, which was also accompanied by choroidal thickening. It is possible that subclinical choroiditis was present at that time as well, although it cannot be proved because no ICGA or LSFG was performed at the time. Furthermore, the fact that all these findings were improved after PSL treatment indicates that the pathological condition involved inflammatory changes.

Previously, two cases of TINU syndrome accompanied with choroiditis have been reported [3, 4]. Like our present case, FA showed fluorescent leakage from the optic disc and peripheral retinal vessels, and ICGA showed scattered hypofluorescent dots. In these two cases, choroiditis was subclinical and not detected by slit-lamp or fundoscopic examination. This suggests the possibility that more patients with TINU syndrome may have choroiditis that would be detected on careful examination with OCT, ICGA, and LSFG.

We previously reported cases of TINU syndrome showing choroidal neovascularization (CNV), which is the most serious complication of TINU syndrome and leads to permanent visual dysfunction [5]. We assume that prolonged choroiditis may induce the development of CNV. The reasonable way to prevent the development of CNV is to accurately identify the presence of subclinical choroiditis that cannot be detected by slit-lamp examination by means of multimodal imaging and then provide adequate treatment.

In this case, the patient’s subclinical choroiditis could be controlled with PSL alone; however, cases in which immunomodulatory treatments were required have also been reported [3, 4]. It is necessary to choose the appropriate treatment individually for each case.

In this case, bilateral hypofluorescent lesions were scattered on ICGA, which were greater in number than fundus white spots or hyperfluorescent lesions on FA. Acute posterior multifocal placoid pigment epitheliopathy (APMPPE), punctate inner choroidopathy (PIC), and birdshot chorioretinopathy (BSRC) are the most common diseases in which bilateral hypofluorescent spots are widely detected with ICGA. However, in APMPPE and BSRC, many bilateral fundus white spots are typically observed at the onset, and in PIC and BSRC, many of the lesions remain as scar lesions during remission, which are different from the present case. Furthermore, the clinical manifestation of uveitis in this patient was panuveitis with retinal exudates and snowball vitreous opacities, which is different from the typical features of APMPPE, PIC and BSRC.

Recently, the concept of “secondary multiple evanescent white dot syndrome (MEWDS)” or”epi-MEWDS” has been proposed [6, 7], which demonstrates quite similar ocular findings to primary MEWDS, usually along with multifocal choroiditis (MFC) or PIC, but rarely with other intraocular inflammatory diseases. Multimodal images in MEWDS include hyperfluorescent lesions on FA consistent with white dots on the fundus, which are hypofluorescent on ICGA and hyperautofluorescent on fundus autofluorescence (FAF), and the disruption of the ellipsoid zone (EZ) and interdigitation zone (IZ) on OCT. In this case, white spots on fundus which were hyperfluorescent on FA and hypofluorescent on ICGA were seen OS, which made secondary MEWDS be considered. However, despite the scattered distribution of bilateral hypofluorescent lesions on ICGA, the essential OCT findings for the diagnosis of MEWDS characterized by the focal disruption of the EZ and IZ corresponding widely with hypofluorescent lesions on ICGA were not seen. Unfortunately, FAF examination was not performed, which is another essential examination for the diagnosis of MEWDS; therefore, we could not confirm that secondary MEWDS occurred in this case. Thus, along with other diseases being ruled out, the patient had interstitial nephritis in addition to uveitis in both eyes; therefore, the diagnosis of probable TINU syndrome was established according to Mandeville's diagnostic criteria [1].

In conclusion, we experienced a case of TINU syndrome complicated by subclinical choroiditis that could be detected with multimodal imaging. Further studies are necessary to determine the frequency of subclinical choroiditis in TINU syndrome and whether it can lead to irreversible visual dysfunction.

### Supplementary Information

**Additional file 1: Supplementary Figure.** OCT image of the left eye at the first recurrence. Swept-source OCT showed subfoveal SRD and choroidal thickening.

## Data Availability

The datasets used and/or analysed during the current study available from the corresponding author on reasonable request.

## Abbreviations

BCVA: Best corrected visual acuity
CNV: Choroidal neovascularization
FA: Fluorescein angiography
ICGA: Indocyanine green angiography
LSFG: Laser speckle flowgraphy
OCT: Optical coherence tomography
OD: Right eye
OS: Left eye
OU: Both eyes
PSL: Prednisolone
SRD: Serous retinal detachment
TINU: Tubulointerstitial nephritis and uveitis

## References

[1] Mandeville JT, Levinson RD, Holland GN (2001). The Tubulointerstitial Nephritis and Uveitis Syndrome. Surv Ophthalmol.

[2] Goda C, Kotake S, Ichiishi A (2005). Clinical features in tubulointerstitial nephritis and uveitis (TINU) syndrome. Am J Ophthalmol.

[3] Caplash S, Gangaputra S, Kodati S, Tuchman S, Srinivasalu H, Sen HN (2018). Treatment challenges in an atypical presentation of tubulointerstitial nephritis and uveitis (TINU). Am J Ophthalmol Case Rep.

[4] Scifo L, Willermain F, Postelmans L, et al: Subclinical Choroidal Inflammation Revealed by Indocyanine Green Angiography in Tubulointerstitial Nephritis and Uveitis Syndrome. Ocul Immunol Inflamm. 2022;30(5):1190–8. 10.1080/09273948.2020.1869267 34191677

[5] Takemoto Y, Namba K, Mizuuchi K, Ohno S, Ishida S (2013). Two cases of subfoveal choroidal neovascularization with tubulointerstitial nephritis and uveitis syndrome. Eur J Ophthalmol.

[6] Essilfie J, Bacci T, Abdelhakim AH (2022). Are there two forms of multiple evanescent white dot syndrome?. Retina.

[7] Yasmine S, Armelle C, Pierre G (2022). Comparison of primary and secondary forms of multiple evanescent white dot syndrome. Retina.

