# Supplementary material for: A case of tubulointerstitial nephritis and uveitis syndrome accompanied by subclinical choroiditis
Source: BMC Ophthalmol. 2023 Oct 20;23:424. doi: 10.1186/s12886-023-03172-0 (PMC10588062; doi:10.1186/s12886-023-03172-0)
Supplement: Supplementary file 1 — Additional file 1: Supplementary Figure. OCT image of the left eye at the first recurrence. Swept-source OCT showed subfoveal SRD and choroidal thickening. [file 12886_2023_3172_MOESM1_ESM.pdf]

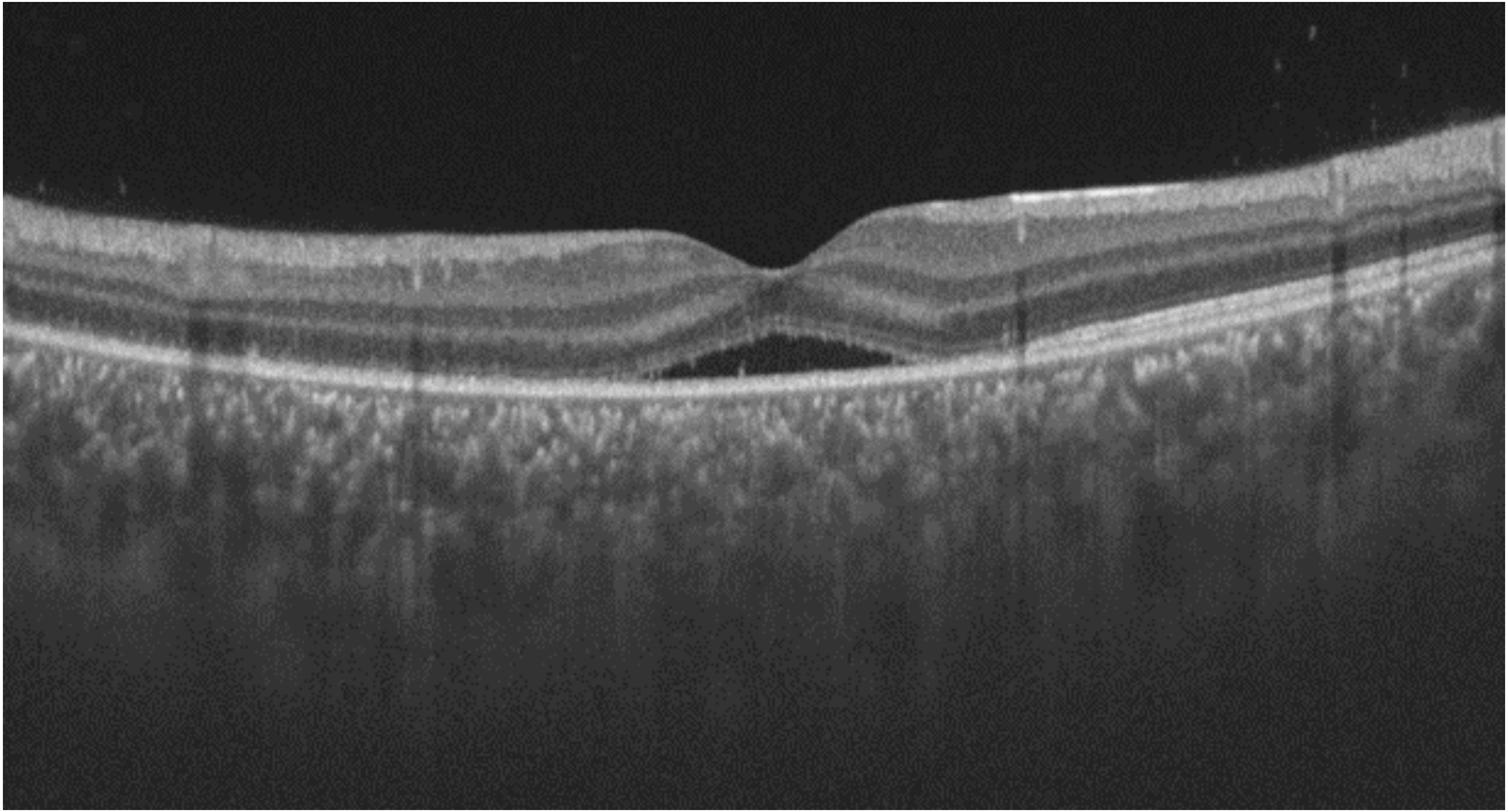

Supplementary Figure. OCT image of the left eye at the first recurrence

Swept-source OCT showed subfoveal SRD and choroidal thickening.
